# Digital Research Skills: Application in Secondary Science Reports

**DOI:** 10.1007/s11165-025-10259-9

**Published:** 2025-05-31

**Authors:** K. N. Blankendaal-Tran, R. F. G. Meulenbroeks, W. R. van Joolingen

**Affiliations:** Freudenthal Institute, Utrecht University, PO Box 9432, 3506 GK Utrecht, The Netherlands

**Keywords:** Digital research skills, Secondary education, Science project report

## Abstract

Digital Research Skills (DRS) are a subset of digital skills that are essential for performing and communicating research in science. This study focuses on the current level of DRS as evidenced by 12th grade pre-university science students in their science project reports (SPRs). 88 SPRs in the fields of the physical sciences were collected. A rubric was constructed using a bottom-up method and served as a coding scheme to systematically assess the levels of DRS demonstrated in students’ SPRs. To ensure validity, the rubric was reviewed by two experts, and inter-rater reliability was assessed. The results demonstrate students’ difficulties in digitally analysing, transforming and visualizing content/data, as well as in writing a research paper using digital tools. Examples are problems with the proper construction of graphs and formulas to an extent that they might confuse the content of the report. The level of DRS as evidenced in students’ reports is generally found wanting. Key deficiencies include inadequate referencing, inadequate figures, and a lack of proficiency in data handling and analysis. The observed deficiencies in DRS in science project reports can lead to significant confusion. We therefore advocate increased attention to DRS in secondary education.

## Introduction

One major goal of secondary science education is to prepare students for an academic career in science and technology. On entering university, these students are expected to demonstrate a number of inquiry-related skills (Masoud & Al Muhtaseb, 2021; Selwyn & Renaud-Assemat, 2020; Wan Yusof et al., 2022).

Research skills in secondary science education are crucial for promoting these skills and a deep understanding of scientific concepts in students. Fischer et. al, defined *research skills* (RS) as a set of “skills and abilities to understand how scientific knowledge is generated in different scientific disciplines, to evaluate the validity of science-related claims, to assess the relevance of new scientific concepts, methods, and findings, and to generate new knowledge using these concepts and methods” (Fischer et al., 2014, p. 29).

In the present day and age, research relies to a large extent on digital technology and therefore requires specific proficiency in this area, (Carretero et al., 2017; Fraillon et al., 2014; Guillén-Gámez et al., 2023; Indah et al., 2022; Kure et al., 2023). An example is handling of large numbers of online databases and scientific journals (Polizzi, 2020). Digital tools also facilitate the writing process and the creation of presentations (Williams & Beam, 2019). Moreover, the application of digital technology is increasingly important in the data collection phase (Shields, 2003; Shopova, 2014). For example, when digitally collecting data using readily available sensors or an online survey, digitally processing large amounts of data and subsequent display of these data is a necessity (Lee & Wilkerson, 2018). We define Digital Research Skills (DRS) as “the subset of digital skills that is essential for performing and communicating research in science”.

The transition from pre-university to a university science study can be challenging in terms of students’ characteristics and motivation (Maddens et al., 2021; Salmela-Aro, 2020; E. Smith & White, 2015), depth of knowledge (De Meester et al., 2020; Di Martino & Gregorio, 2019) and academic writing skills, such as information literacy skills (Julien & Barker, 2009; Salisbury & Karasmanis, 2011; Wollscheid et al., 2021).

The continuous appearance of reports in literature highlighting freshman students’ deficiencies in the area of DRS underscores the need for attention in this domain. To mention a few examples, Youssef et al. (2022) have reported the digital divide in France and its effect on students’ academic performance. Masoud & Muhtaseb (2021) concluded that students need to improve formatting and punctuation in their reports. Abdullah et al. (2011) observed that 67% of first-year academic students in Malaysia were somewhat competent in spreadsheet skills and 12% of were not competent at all. Calvani (2012) enlarged this gap in DRS and found that when upper secondary school students in Italy used a spreadsheet during experimental models, they obtained less than 40% correct answers. Akuegwu and Uche (2019) affirmed this gap in Nigeria and found that whereas students’ reading, presentation, communication and information-gathering skills were adequate, especially data analysis was found wanting. Other international research, including in the U.S.A, Canada, and the Netherlands, confirmed this problematic application of skills, such as spreadsheet skills, information-seeking skills and graph construction skills among students in secondary education (Bobkowski & Etheridge, 2023; McHugh et al., 2021; Meelissen et al., 2014; J. K. Smith et al., 2013; Walraven et al., 2008).

Ten years after Abdullah’s suggestion to incorporate DRS—such as word processing and spreadsheet skills—into curricula, the issue remains largely unresolved. This is reinforced by the conclusions of reports on challenges for curriculum developments concerning both research skills and digital literacy skills (Erstad et al., 2021; Lacson & Dejos, 2022; Polizzi, 2020; Vieno et al., 2022). Some national secondary education syllabi provide guidelines on the development of these skills. However, concrete demands concerning the level of these skills are often lacking. In the Netherlands, the context of this study, the examination program states that students must be able to gather, process and analyse data digitally, without further indications on how to assess these skills (CvTE, 2021a, b). It may thus come as no surprise that university teachers are concerned about the difference in DRS between pre-university and academic science education. In a recent interview study, it was found that university teachers in the natural sciences have concerns about the level of DRS demonstrated by students entering university, as exemplified by poor performance in referencing as well as analysing, transforming and visualising data (Blankendaal-Tran et al., 2023).

One key assignment in secondary science education that relies on the application of DRS is the science project. This process can be seen as a complex integrative research activity that stands at the crossroads of several domains, such as disciplinary knowledge, critical thinking and communicative and discourse related abilities (Elander et al., 2007; Kruse, 2013). A popular assessment method is to have students write a report on a research project. These science project reports (SPRs) are regarded as both a useful assessment tool in education and as a valuable means of promoting conceptual learning, both in higher and secondary education (Graham et al., 2020; Haines, 2021; Hort, 2020; Zeegers & Giles, 1996).

Given this situation, it is unfortunate that it is often not clearly defined at what level DRS need to be addressed in secondary science education. It is also often not clearly defined to what extent students need more practice to prepare them for the transition to a tertiary science study. The present study addresses the level of DRS in secondary science education (as: fully correct, partially correct, or incorrect), as evidenced in students’ final SPR. This is an important step towards bridging the gap in digital research skills from secondary science education to an academic study.

The following research question will be addressed: What is the current level of digital research skills (DRS) of pre-university science students as evidenced in their research reports?

To answer this question, we designed a rubric to structurally analyse DRS. We will then use this DRS rubric as coding scheme for content analysis on students’ science projects reports.

## Theoretical Framework

### Operationalizing DRS

Before being able to assess students’ DRS, we need to operationalize the concept into a form that allows us to glean its level from student work. In the review study of Van Laar et al. (2017), the fusion of digital literacy skills and 21st-century skills is called 21st-century digital skills. However, the review by Van Laar et al. (2017) focuses on general skills, such as communication, collaboration, and creativity. Guillén-Gámez (2020) used the term ‘digital research skills’ to refer to the integration of ICT tools, but describes these digital competencies for teachers across various fields in higher education.

Returning to our definition of DRS as “the subset of digital skills that are essential for performing and communicating research in science”, we note that a lack of proficiency in these skills thus negatively impact the quality of the research itself or the reporting on the research. To give an example, when a scatter plot only shows the measuring points, legend and title, the resulting graph is essentially unintelligible. Students may know they need to add axis titles to the graph and what should be mentioned in the axis titles (which is a research skill), but they may lack the DRS to actually implement this. Alternatively, consider the search for information or the use of software to process large-scale representative data sets. This is a part of DRS in the sense that a lack of this skill can lead to erroneous or inaccurate conclusions or representations (Guillén-Gámez et al., 2020).

The distinction between research skills versus DRS on the one hand, or digital literacy skills versus DRS on the other, is not trivial. For example, even though scientific writing is itself a research skill and not part of DRS per se, many of the related (and essential) skills are DRS. The use of formulas is also not a digital research skill. However, the ability to effectively use the formula editor in a word processor is. If this skill is lacking, formulas can easily be misinterpreted, as will be shown further below.

By combining existing frameworks for research skills and digital literacy skills, a guiding framework of DRS was constructed (Blankendaal-Tran et al., 2023). This DRS framework incorporates seven main categories: (1) Browse, search and filter information, (2) Gather, measure and collect digital content/data, (3) Determine the accuracy and validity of sources/methods, (4) Structure, manage and protect digital content/data, (5) Analyse, transform and visualise content/data digitally, (6) Write a research paper using digital tools and (7) Share and present content/data.

### Assessing DRS

In the current study we are interested in the application of DRS by pre-university students in their final year, as exemplified by the report on their mandatory Science Project Report (SPR) in their final year. We studied a represented sample of these SPRs for evidence of the application of DRS.

There is a relative dearth of studies that assessed DRS or even digital literacy skills using student products, such as SPRs. One exception is Parra and Calero (2019), who investigated the contribution of the Automated Writing Evaluation (AWE) program to measure the learners’ writing skill. AWE appeared to help improve learners` writing quality due to its fast and individualized feedback with explanations of grammar, spelling, sentence and word usage, contributing to learners’ autonomy. Furthermore, Williams and Beam (2019) found that, in K-12 educational settings, students’ writing skills could be measured and improved using a writing rubric. This was made possible due to the use of digital technologies and web-based learning environments. However, this only involved short digital writing assignments.

### Use of Rubrics in Assessments

Rubrics are often used in the assessment of student learning at all levels (Wolf & Stevens, 2007). Gallardo (2020) describes the use of rubrics as a powerful tool to measure knowledge, skills and attitudes, including the advantages and future challenges. Rubrics have shown to be particularly effective for improving the assessment of complex tasks or projects, and for promoting the development of higher-order thinking skills (Jonsson, 2014; Reddy & Andrade, 2010). Rubric-based studies that assess science reports mainly mark structure and coherence of the content, and the use of relevant references or organizational criteria, such as numbered table of contents and page numbers (Cantera et al., 2021; Rakedzon & Baram-Tsabari, 2017). Rubrics are also used to assess digital skills, such as the format of figures, tables and equations in general (Wright et al., 2022). Recently, Wan Yusof et al. (2022) used a rubric to measure practical and scientific writing skills in secondary student projects’ final reports. One of the parameters in their assessment can be marked as a part of DRS: ‘Students include appropriate symbols, labels, units, and significant figures during data collection.’ Student reports were also assessed on the use of appropriate tables/figures, labelling of tables/figures, calculations and the use of citations and references, to the latter through an online plagiarism check. These examples indicate that rubrics can be used effectively in assessing different aspects of SPRs. The studies by Wan Yusof et al. (2022), Rakedzon and Baram-Tsabari (2017), and Cantera et al. (2021) demonstrate that rubrics can be used as a reliable tool for assessing the application of scientific writing skills and can be adapted for evaluating other genres as well. Inter-coder reliability achieved an acceptable level of agreement, with scores in the 0.70 range.

### Assessing DRS in SPRs

In a digitally submitted Science Project Report (SPR), e.g., in PDF format, not all DRS can be positively identified. For example, it remains uncertain whether the student has updated the numbering of figures automatically or manually. We therefore cannot distinguish in our analysis between correctly displayed or automatically updated page or figure numbers. Furthermore, a digital SPR does not evidence the manner in which a student searched for literature and sources. It is only possible to see what types of sources are used and to what extent they are reliable. These aspects of DRS are thus clearly not identifiable in a PDF version of an SPR.

We refined the DRS concepts during many onsite, lengthy discussions between the authors. The following examples from our study illustrate aspects of DRS and the distinction between DRS and digital skills or research skills. We consider the issues covered in these examples to be mainly due to a lack of *digital* research skills. Four main categories are described: diagrams, formulas, use of sources, and general text formatting.

#### Example 1: Diagrams

**Fig. 1:**
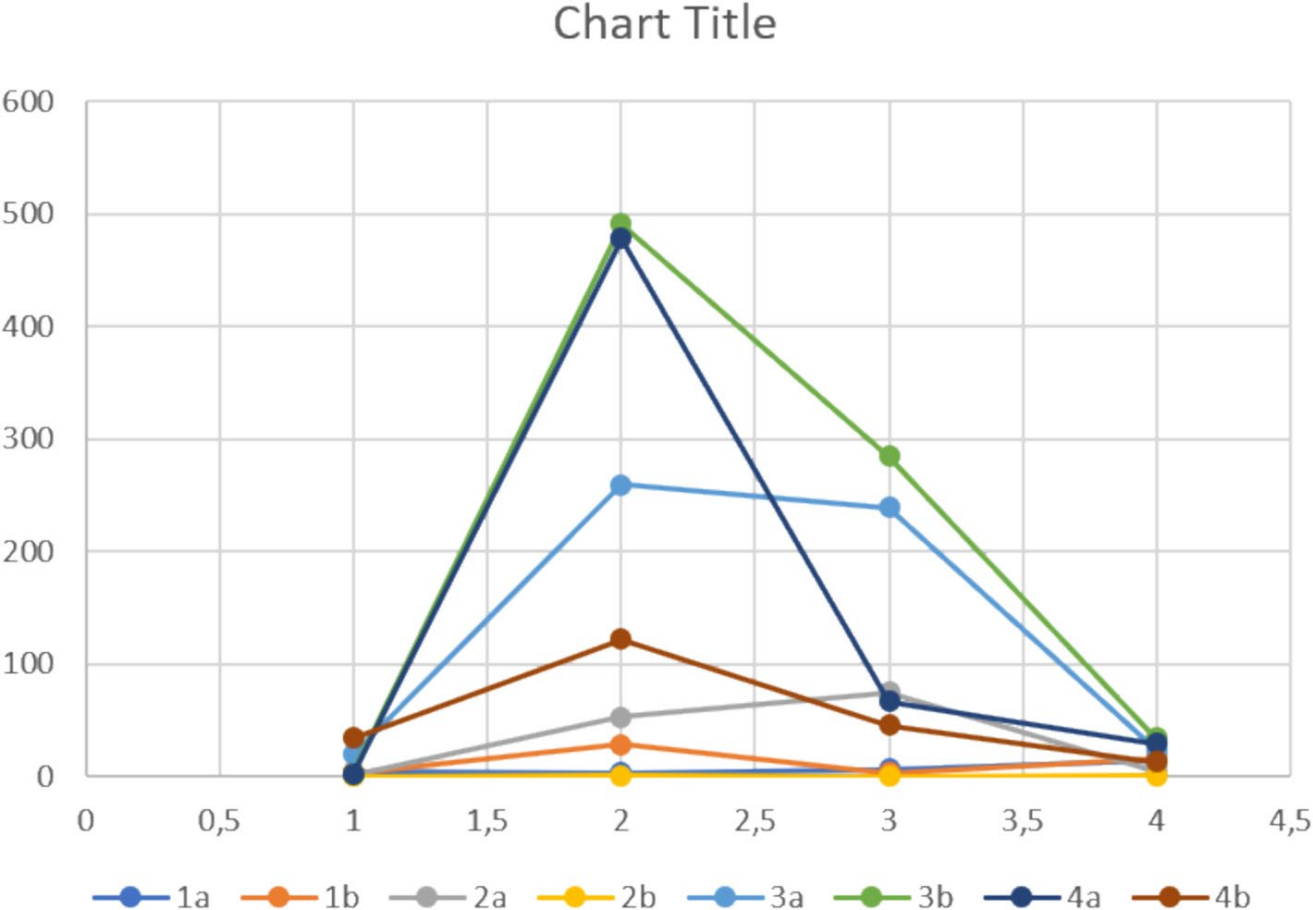
DRS is not properly applied in Excel to visualise the data. The voltage (U) of 4 different types of solar cells, each of an old (**a**) and new model (**b**), is measured under four different conditions. Excel recommends this type of scatter chart and automatically inserts a chart title, a contour and half values on the horizontal axis, which have no meaning. However, additions such as axis titles with quantities and units are not automatically inserted. Application of DRS would involve not just clicking on the recommended graph, but using one’s own judgment in selecting and formatting a graph

Context:

In Fig. 1, the voltage (*U*) of 4 different types of solar cells, each of an old (a) and new model (b) is shown. *U* is measured under four different conditions (low and high temperature, with a barrier and with an extra electrolyte).

Issues:

Based on the data entered, a spreadsheet program such as Excel recommends the type of scatter chart shown in Fig. 1. At first sight, the figure seems in order. There is a clear distinction between the lines, however, with closer scrutiny, DRS appear insufficient. The graph falsely suggests a correlation between the values on the horizontal axis and the measurement points, while this is not the case. Excel also automatically insert a chart title, a contour, and axis values to 1 decimal place on the horizontal axis, which is meaningless. However, meaningful additions, such as axis titles with quantities and units, are not included automatically.

**Fig. 2:**
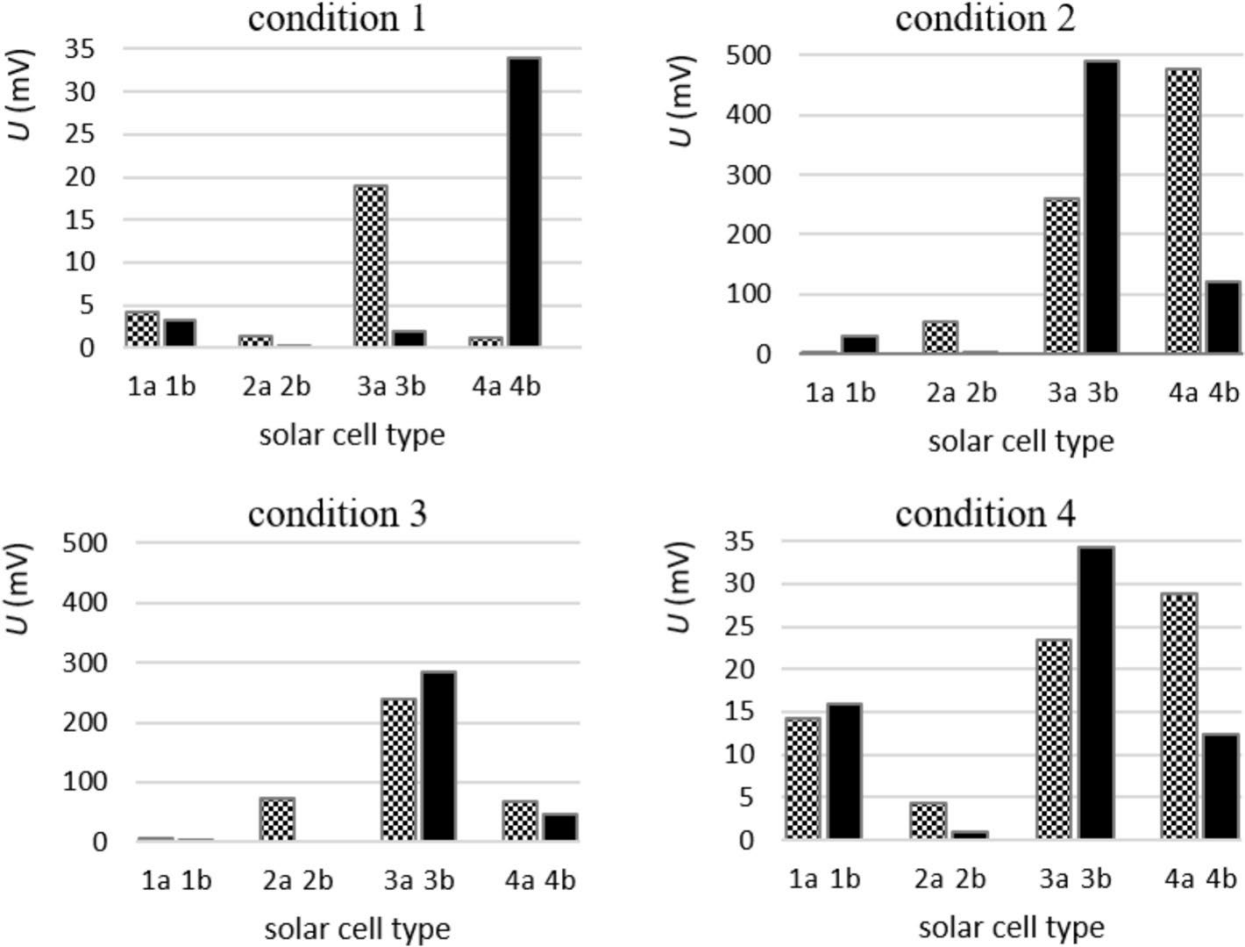
DRS is properly applied in Excel to draw four comprehensive bar charts. The voltage (U) of 4 different types of solar cells, each of an old (**a**) and new model (**b**), is measured under four different conditions

Correct application of DRS:

Application of DRS would involve not just clicking on the recommended graph, but using one’s own judgment in selecting a representation and then using the spreadsheet to produce just that. Since there is no correlation between the four conditions in this example, four different bar charts would present the results more comprehensively, as shown in Fig. 2. Figure 2 also applies DRS for formatting graphs.

#### Example 2: Formula editing.

Context:

An imaginary formula is used to illustrate this, η_q_ = I_d_^n-2/I_c_^2-n.

Issue:

Formulas which are made without a formula editor may create ambiguity. In this case, the formula can be interpreted as ${\eta }_{\mathrm{q}}={{I}_{\mathrm{d}}}^{n}-\frac{2}{{{I}_{\mathrm{c}}}^{2}}-n$ or as ${\eta }_{\mathrm{q}}=\frac{{{I}_{\mathrm{d}}}^{n-2}}{{{I}_{\mathrm{c}}}^{2-n}}$.

Correct application of DRS:

In this case, ambiguity would have been avoided, if the formula editor in the word processor had been used.

#### Example 3: Use of references

Issues:

When students have mastered research skills such as searching and using scholarly articles, they are unable to reference them according to a guideline, such as APA. For example, a student’s text states: ‘The charge efficiency is influenced by multiple factors (Sciencedirect, 2015).’ A student who has mastered DRS would refer to the author(s) rather than a database. Students may also use footnotes at the end of each page, such as:

27 Smith, B., & Lin, H. A. (2020). State of charge. *Journal of ….*

Correct application of DRS:

A student who has mastered DRS would utilize the digital features within the software, for instance in Word, to manage, generate and customize the reference list automatically at the end of the document. Additionally, when changing the order of the sources in the text, the source list could be updated automatically.

#### Example 4: General formatting

Issues and solutions:

Students mastering research skills may use undefined quantities in the text, which confuses the reader. This happens, for example, in situations where students type Greek or non-capital letters at the beginning of a sentence. Word automatically capitalizes *η* as *H* and *a* as *A*. Students should correct this using digital skills. Another possible indication of a lack of DRS is the notation of quantities and units, without italicization, symbols and super-/subscripts. A student who has mastered DRS would opt for *T* (⁰C) and *a* (m/s^2^), instead of ‘temperature in degrees Celsius’ and ‘acceleration in meters per second squared’.

Students who have mastered DRS would also add a caption, including automatic numbering, to understand a graph or table.

Note that the insertion of a figure itself is not considered to be a part of DRS: the figure may have been inserted by physical cutting and pasting. It is the captioning, alignment, proper margins, use of a high-quality file, removing/avoiding a watermark, etc., that constitute DRS.

## Method

As described in Sect."Use of rubrics in assessments", rubrics provide a standardized way to measure skills across predefined criteria. Therefore, this study employs a structured DRS rubric as a coding scheme to systematically assess the applied DRS and their corresponding levels in students’ SPRs.

### Sample

**Fig. 3:**
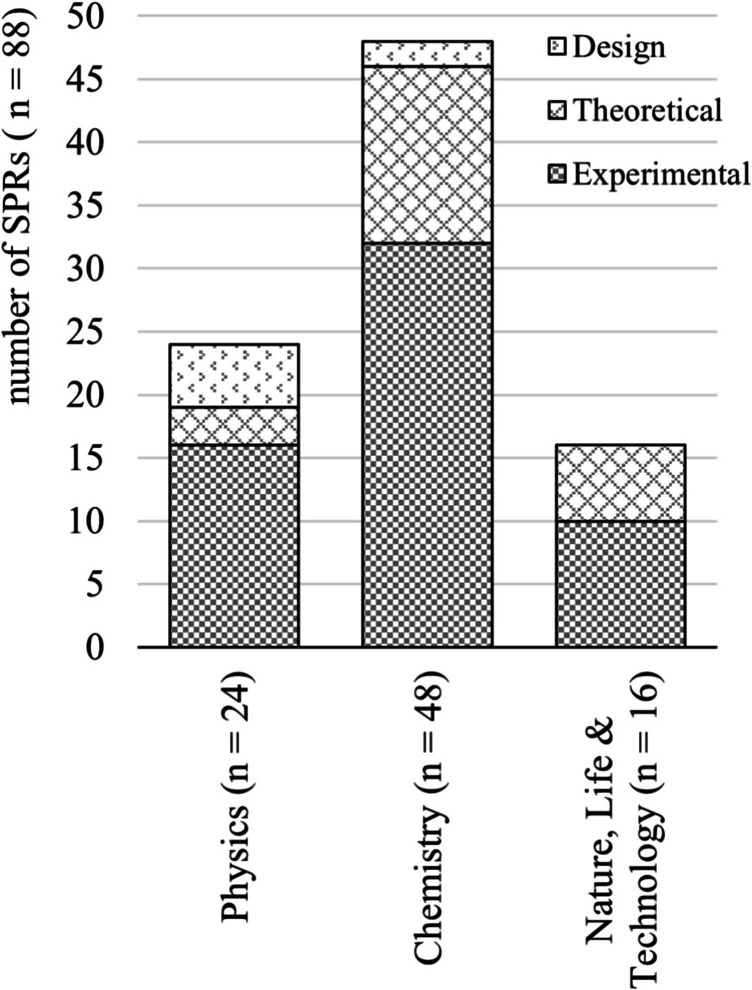
Distribution type of SPRs across physics, chemistry and NLT

A total of 88 research reports by 11 th and 12 th-grade pre-university students from a mix of schooling systems was collected in the period from March 2021 to May 2022. This allowed us to collect SPRs from exam students from across the country over two consecutive school years. The collected reports describe research in physics (*n* = 24), chemistry (*n* = 48) and nature, life and technology (*n* = 16). The reports describe experimental research (*n* = 58) and theoretical research (*n* = 23), as well as technical designs (*n* = 7), see Fig. 3.

The research reports were written in the context of the final examination in the Dutch educational system. As part of this exam, all pre-university students in the Netherlands are required to write such an SPR. These students are expected to conduct a research project and subsequently write a research paper. For the collection of the SPRs (A) we approached 100 science teachers, using social media and newsletters. The teachers held positions in different schools throughout the country. To avoid bias, the teachers were asked to provide SPRs from all students that they had supervised, that all SPRs would be anonymized, and that evaluation or rating was not part of the study. Both teachers and students did not know in advance how and on what the reports would be analysed. A total of 27 teachers responded. (B) Before sharing the SPRs, these teachers collected informed consent signatures from the students. The students’ SPRs were digitally submitted by 13 physics and 14 chemistry teachers; 7 of the total group of 27 teachers also teach the interdisciplinary course NLT.

(C) After downloading the submitted files, student, teacher and school names were removed from the reports, as were contact information, logs, prefaces and acknowledgements, to ensure anonymous processing. Also, as an extra check, we used an online plagiarism detection tool to establish the absence of plagiarism for each SPR. No SPR had to be excluded after this check. The majority of SPRs were submitted as PDFs, others as Word files. We pseudonymized all SPRs, added a code to the title page and converted the Word files to PDF before analysis.

### Data Analysis

The previously mentioned DRS framework (Blankendaal-Tran et al., 2023) was used as the basis of the rubric used for analysis. The rubric comprises five of the main categories from that framework: 1 ‘Browse, search and filter information’, 2 ‘Gather, measure and collect digital content/data’, 3 ‘Determine the accuracy and validity of sources/methods’, 5 ‘Analyse, transform and visualise content/data digitally’ and 6 ‘Write a research paper using digital tools’. The fourth (Structure, manage and protect digital content/data) and seventh (Share and present content/data) categories of the DRS framework are not usually detectable in a PDF or Word-processed version of an SPR, and have therefore not been included in the rubric.

The rubric was constructed following a bottom-up method, using an initial sample of five randomly selected student SPRs in different domains of secondary school science. These five SPRs include: one design study in physics, one experimental study in physics, two experimental studies in chemistry and one combination of a design and experimental study in chemistry. We started top-down from the five main DRS categories and operationalized these by forming the subcategories bottom-up, see Table 1. After analysing the five SPRs on DRS, the identified DRS characteristics were categorized on the basis of the aforementioned DRS framework. Since no new subcategories needed to be added during analysis of the fifth SPR, we assumed that data saturation had been reached by that point.

**Table 1:** Overview of the DRS main categories and subcategories, based on the DRS framework (Blankendaal-Tran et al., 2023). * Mandatory items during assessing

	**DRS categories**	**Subcategories**
1	Browse, search and filter information	• Number of digital resources * • Use of scientific sources *
2	Gather, measure and collect digital content/data	• Digital measuring tools
3	Determine the accuracy and validity of sources/methods	• Referring, quoting and paraphrasing *
5	Analyse, transform and visualise content/data digitally	• Digitally processing large amounts of data • Selection diagram • Display of data from different types of measurements • Using software with calculation function • Multiple data sets displayed in one diagram • Display graphs • Axis titles • Trendline • Legend • Error bars • Formatting graphs • Display tables • Formatting tables • Display pictures/images • Reaction equations • Drawing structural formulas • Programming/Technical drawing
6	Write a research paper using digital tools	• Page numbering * • Images/figures/tables numbering • Table of contents * • Insert captions * • Reference list * • Styles and headings * • (Non) Italics • Sub-/Superscript • Symbols • Listings • Line spacing * • Text wrapping * • Layout, alignment and margins * • Header and footer • Representation research design • Formula/calculation

We consulted two experts in the field to review the rubric and ensure that it accurately reflects the essential components of DRS. Consensus was reached through weekly discussions with the authors. These meetings addressed the inclusion criteria, structural organization, and specific rubric content necessary to accurately assess DRS. The final rubric consists of 5 categories and 37 subcategories and is given in Appendix - Table 2. All subcategories were assigned three mastery levels (score 2, 1 and 0) and associated criteria according to ascending level. Score 2 is obtained when a DRS is applied correctly, score 1 when it is applied partially correctly and score 0 when applied incorrectly.

To be more specific, skills in DRS categories 1 ‘Browse, search and filter information’ and 3 ‘Determine the accuracy and validity of sources/methods’ are considered mandatory in an SPR. Scores for the subcategories are always assigned here. In category 6 ‘Write a research paper using digital tools’, only part of the subcategories is considered mandatory, see Appendix - Table 2. In categories 2 ‘Gather, measure and collect digital content/data’ and 5 ‘Analyse, transform and visualise content/data digitally’, scores are only assigned when the skill has been applied. During the testing phase, the developed rubric was evaluated by the co-authors.

### Coding and Analysing SPRs

**Fig. 4:**
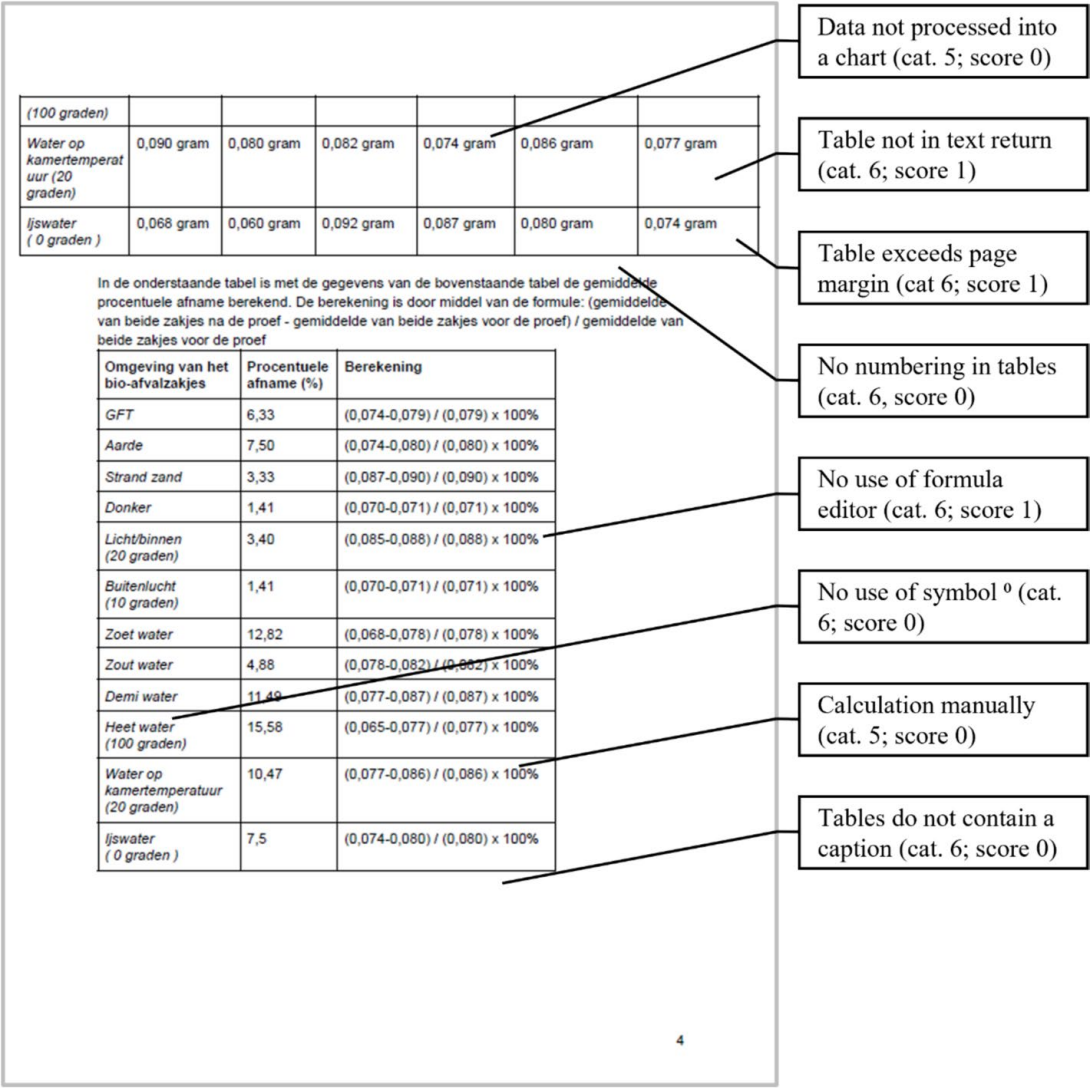
Example 1 of rating an SPR (DRS category number; obtained score) (Cat. 5: Analyse, transform and visualise content/data digitally. Cat. 6: Write a research paper using digital tools)

**Fig. 5:**
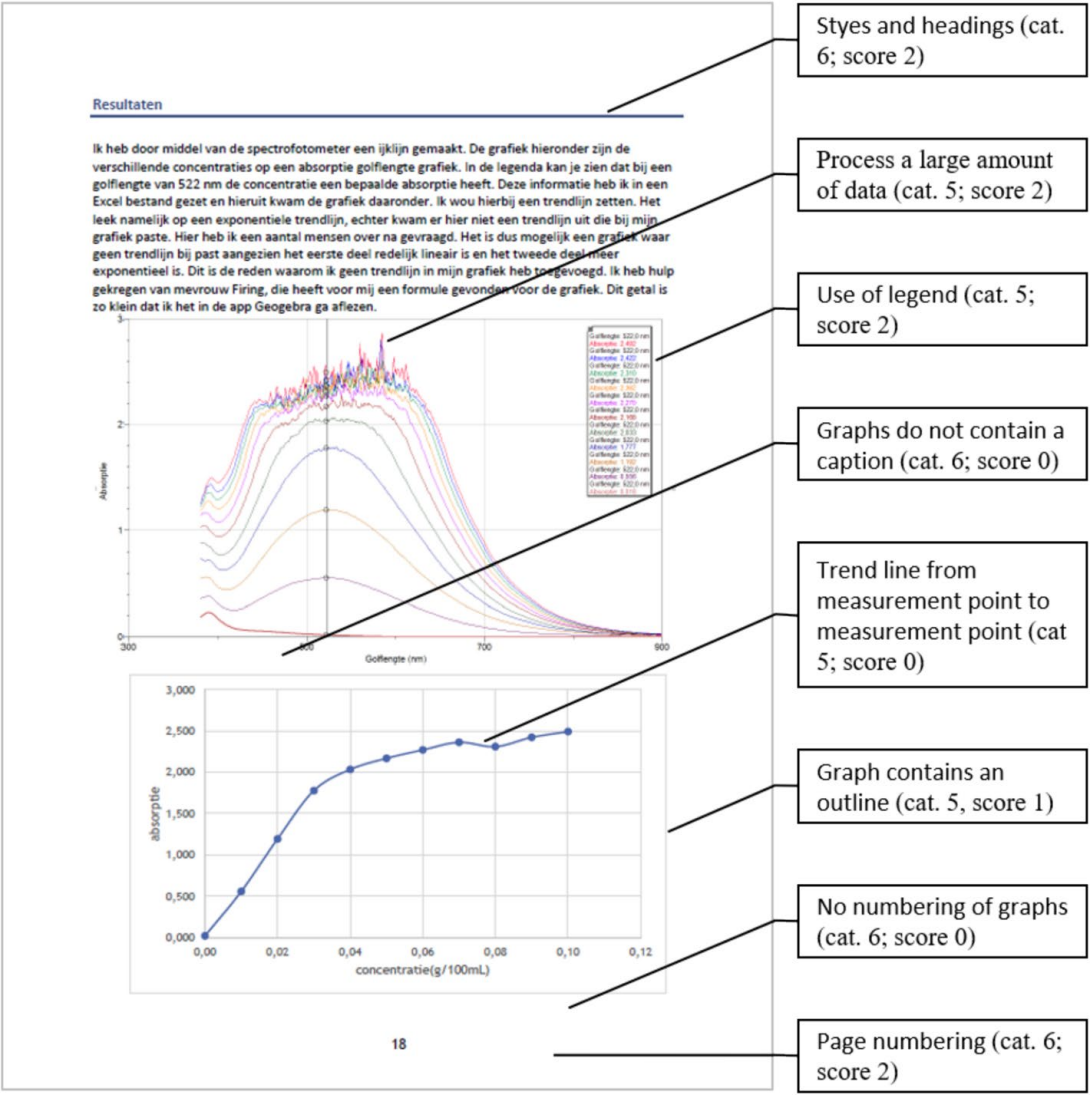
Example 2 of rating an SPR (DRS category number; obtained score) (Cat. 5: Analyse, transform and visualise content/data digitally. Cat. 6: Write a research paper using digital tools)

The SPRs were coded and rated according to the rubric for DRS, see Appendix - Table 2. The parameters for scoring were clearly defined within the rubric, ensuring consistency and reliability. Duplicate coding was performed on 5 SPRs. From the total of 190 items to be assessed, 2 items scored differently, corresponding to 99% concordance. Each SPR was fully analysed in terms of observed DRS as well as in terms of the skill level as evidenced in the report. For example, digital measurements were gauged under DRS Category 2, ‘Gather, measure and collect digital content/data’. For data that are collected using automated measurement via a computer, e.g., online survey/simulation/sensors/applet/Arduino, a maximum of 2 points can be scored in this category. When a computer is used, but measurements are not automated, e.g., in an online video interview, 1 point is scored. When data are gathered manually, there are no scores given. If no empirical data are collected (theoretical SPRs), no scores are assigned in this category. Examples of scoring are given in Fig. 4 (Example 1) and Fig. 5 (Example 2).

Converting Word files into PDF leads to the loss of certain document data, including the number of edits and editing time; automated features, such as the use of styles and headings; and inserting figures using text reflow. However, this data loss did not result in any analysis issues in terms of DRS. For example, the use of styles and headings can also be identified in PDF by clicking on the relevant sub-headings in the report’s table of contents.

## Results

**Fig. 6:**
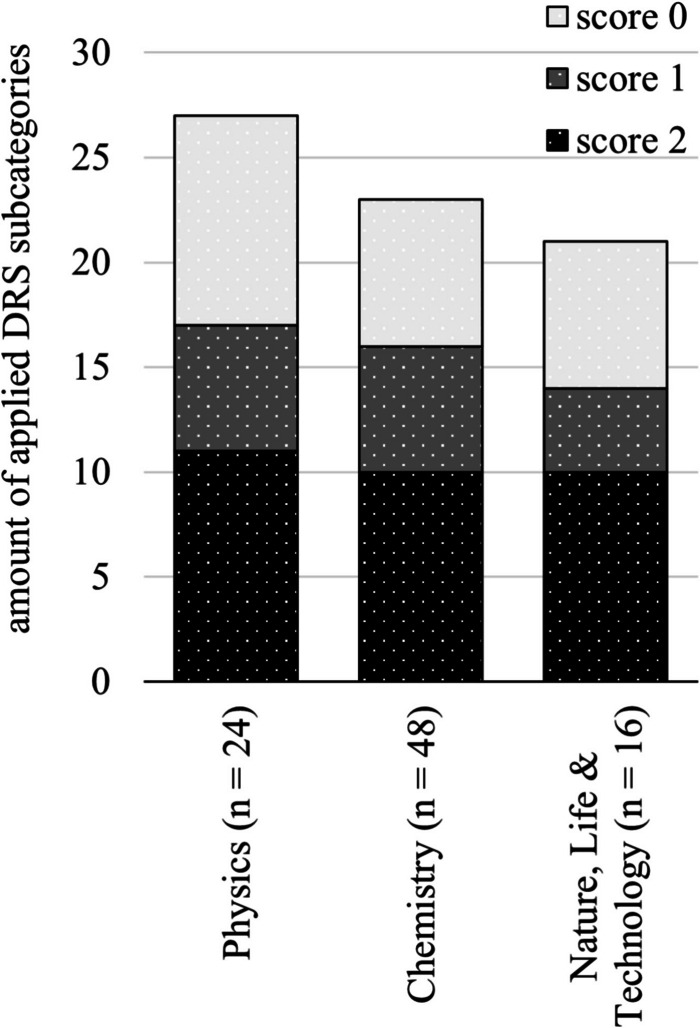
Number of applied DRS subcategories across physics, chemistry and NLT

Figure 6 shows that for SPRs in physics, 27 out of 37 subcategories are applied, while SPRs in chemistry and NLT score somewhat lower in this respect (23 of 37) and (21 of 37), respectively. In physics, an average of 11 out of 28 of the DRS subcategories are applied correctly, as compared to 10 for chemistry and 10 for NLT (score 2).

Students apply DRS slightly more often (both correctly and incorrectly) in physics research reports, compared to chemistry and NLT. On average, 10 out of 28 subcategories are applied incorrectly (score 0) in physics and 7 in chemistry and NLT.

**Fig. 7:**
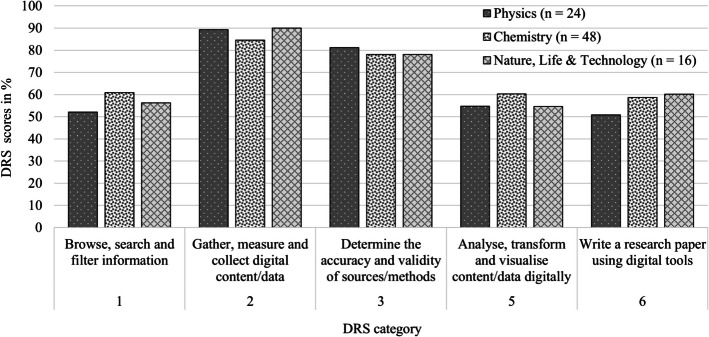
DRS scores in % divided over the DRS categories across physics, chemistry and NLT

Figure 7 illustrates scores as percentage relative to maximum scores per DRS category for physics, chemistry and NLT. For example, with the physics SPRs 25 points were assigned to skills in ‘Gather, measure and collect digital content/data’, with a potential of 28 points if correctly implemented in all 14 SPRs, resulting in an 89% score. This figure gives no information about how often a digital research skill was applied, but rather the extent to which it was applied correctly. More than 50% of the potential points were achieved in all categories.

In particular, for DRS Category 3, ‘Determine the accuracy and validity of sources/methods’, more than 75% of the points were obtained. There were no significant differences in correct DRS application between physics, chemistry and NLT subject across the categories. See Appendix - Table 3 for the main findings by category and subcategory.

### Use of Sources and Referencing

**Fig. 8:**
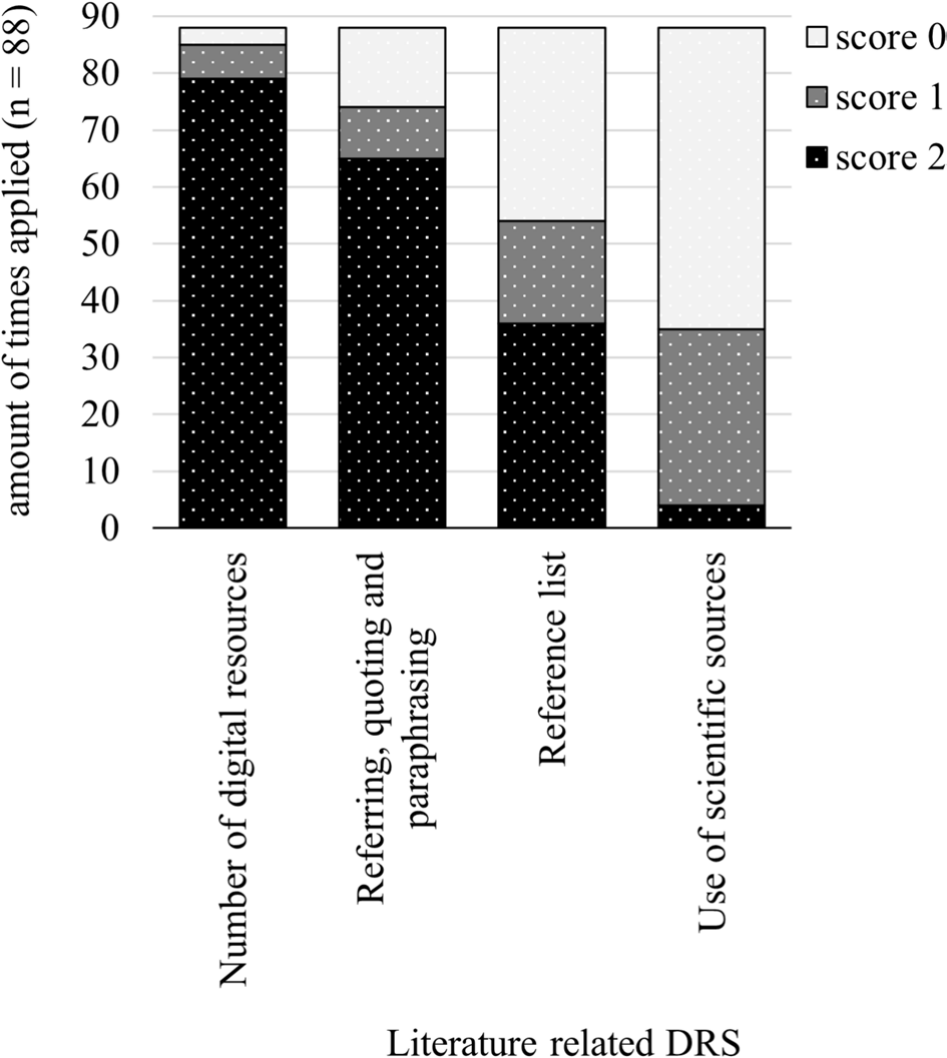
Application of literature-related DRS

Figure 8 shows that 79 out of 88 SPRs contain sufficient amount of sources to support the line of reasoning, e.g., their physics textbook, governmental and journal websites. However, these are mainly Internet sources of sometimes questionable quality, such as forums and websites of companies and institutes (84/88). Peer-reviewed international journals were used in 31 SPRs, with more than half of the sources being scientific peer-reviewed in 4 SPRs.

Regarding referencing conventions, APA or IEEE styles are correctly applied in 36 out of 88 SPRs (score 2). However, in 18 SPRs, internet pages are incorrectly listed with URLs of databases instead of author’s names and titles (score 1). In 34 SPRs, bibliographies are incorrectly formatted. This includes sources listed by chapter or as footnotes by page, or URLs listed consecutively without authors’ names and titles (score 0).

The references in the main body text are correctly applied (APA/IEEE) in 65 of the 88 SPRs. However, in 9 SPRs, referencing is inadequate after a claim, assertion, or fact (score 1), and in 14 SPRs, sources are not referenced at all (0 points).

### Gather, Measure and Collect Digital Content/Data

See Fig. 9

**Fig. 9:**
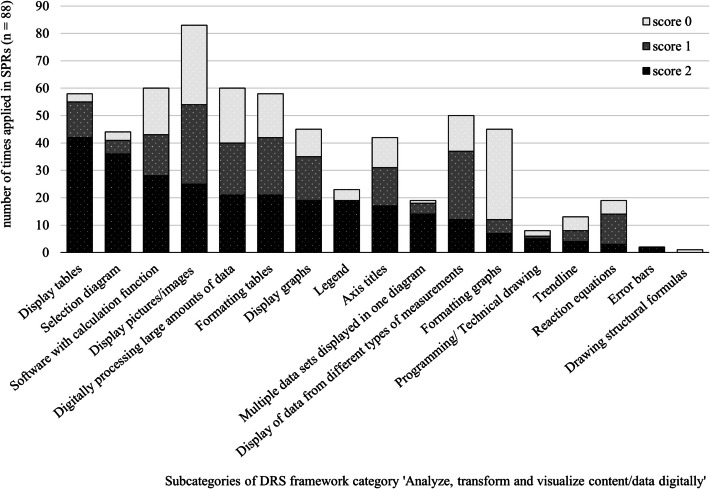
Number of times application of DRS ‘Analyse, transform and visualise content/data digitally’ (category 5)

### Analyse, Transform and Visualize Content/Data Digitally

In Fig. 9, the obtained scores in DRS Category 5, ‘Analyse, transform and visualise content/data digitally’ are shown, ordered according to descending level 2. A score of 2 points is obtained in over 50% of the studied SPRs in the following six subcategories; displaying data in a Table (42 of 58), selection of a proper diagram (36 of 44), display data in a graph (26 of 44), adding a legend (19 of 23), display multiple data sets in one diagram (14 of 19) and the use of programming/technical drawing (5 of 8). For the remaining 11 subcategories, more than half of the SPRs in which they are applied, the DRS are applied either partially (score 1) correct or incorrect (score 0).

Figure 9 illustrates that inserting an image is the most frequently used digital research skill in the 88 SPRs. However, in 29 of the 83 SPRs where this subcategory is applied, figures appear either too dark or too small, resulting in unclear arrangements or text (score 1). An equal number of reports contains watermarked figures or include terms that have not been explained in the text (score 0). Interestingly, in 5 SPRs, the text is not supported by images at all.

Regarding inserted tables, the most common issues include inconsistent text alignment or column headings that are indistinguishable from the rest of the text (score 1, *n* = 19/58). We also see that columns and rows are not in proportion or the number of significant figures is not consistent (score 0, *n* = 16/58).

The majority (60 of 88) of the SPRs contains collected quantitative data. To process data, most SPRs show the use of software, such as Excel (score 2, *n* = 28/60). In a small number of SPRs, charts were made using learning science software, such as Coach 7 (score 1, *n* = 15/60) and there are also reports where the data obtained are processed manually (score 0, *n* = 17/60).

When data from different types of measurements are collected, more than half of the reports use one type of diagram to represent the data (score 1, *n* = 25/50). Surprisingly, in 13 reports, data from different types of measurements are only presented in a table and not processed into a diagram at all (score 0).

In 45 out of 88 SPRs the data are presented in a chart. In 19 of these reports, graphs are displayed correctly (score 2). In 16 out of 45 reports, the graph is displayed almost correctly, with errors including, for example displaying axis values inconsistently or not using secondary grid lines, making the measurement points difficult to read (score 1). The remaining 10 reports scored 0 points for inadequate graph presentation, such as only drawing a line without measurement points. Of the 45 SPRs with graphs, 38 scored 0 for graph formatting, e.g., because the graph contained a title above it instead of a caption (score 0) or scored 1 point because of a dark grey outline around the graph. These two elements are added by default by Excel and were not removed by the students.

In 14 out of 42 SPRs that contain a scatter graph, at least one of the axis titles is incorrect, e.g., it contains a quantity but no unit or an incorrect unit (score 1). Also, in 11 out of 41 reports containing a graph, the axis titles are missing (score 0). When a pie chart was used, axis titles are not considered as required in this study.

Finally, in 13 out of 58 SPRs involving an experiment, the student added a trend line. A portion did not have the trend line drawn through the origin when it should have been, or did not update the trend line formula with the correct quantities or units (score 1, *n* = 4/13). In 5 of 13 reports, a line was drawn from measurement point to measurement point instead of a line drawn through the measurement points (score 0).

### Write a Research Paper Using Digital Tools

**Fig. 10:**
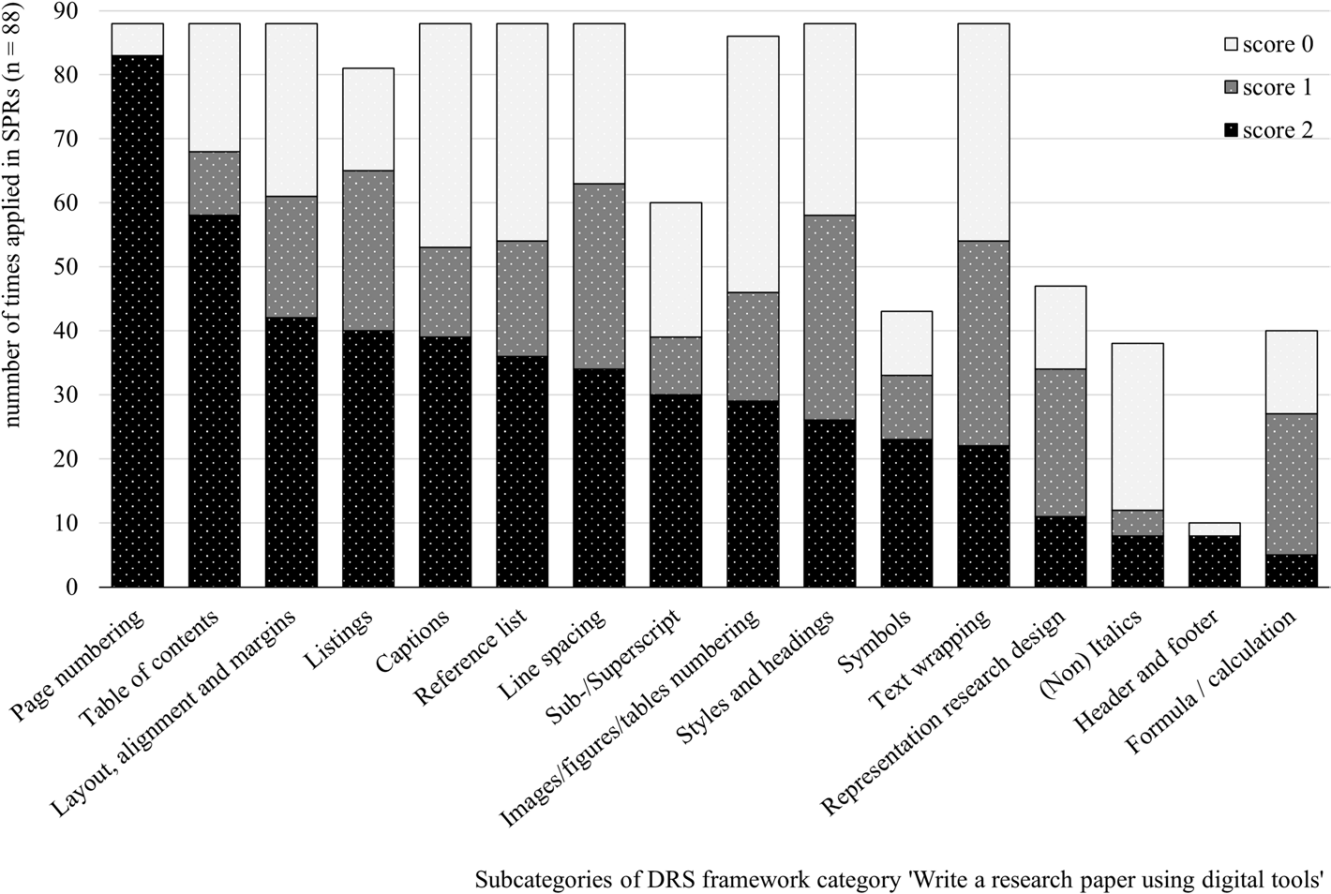
Number of times application of DRS ‘Write a research paper using digital tools’ (category 6)

In Fig. 10, the obtained scores in DRS Category 6, ‘Write a research paper using digital tools’ are shown. Zooming in on the skills in this category, we see that more than half of the SPRs score 2 points on the following two subcategories; reports mainly contain page numbering (83/88) and a table of content (58/88).

Focusing on the remaining 14 subcategories, inserted figures and tables do not always contain correct numbering (score 1 and 0; *n* = 57/86) and a caption (score 1 and 0; *n* = 49/88). Furthermore, text is sometimes inappropriately aligned (score 1 and 0; *n* = 46/88), no use is made of styles and headings (score 1 and 0; *n* = 62/88), text wrapping (score 1 and 0; *n* = 66/88), line spacing (score 1 and 0; *n* = 54/88), use of listings (score 1 and 0; *n* = 41/81) and symbols (score 1 and 0, *n* = 20/43) when needed. Physical quantities and chemical formulas are very often used without applying italics (score 1 and 0; *n* = 30/38) or sub- and superscripts (score 1 and 0; *n* = 30/60).

Students make little use of the formula editor in the word processor. Only 5 SPRs show the correct use of the formula editor, while 22 out of 40 show flaws (score 1). A common mistake is that both magnitudes and units are automatically italicized when using the formula editor in Microsoft Word. In some reports this is not corrected by un-italicizing the units. It is also problematical that a number of students do not use the formula editor at all (score 0, *n* = 13/40).

## Discussion

### Research Question

Our research question was: What is the current level of digital research skills (DRS) of pre-university science students as evidenced in their research reports?

Our results show that all secondary science students in our study apply digital skills to write a research paper. However, the extent to which DRS are applied varies considerately.

For example, all Science Project Reports (SPRs) show that students are able to conduct an online search for digital sources. However, articles from peer reviewed international journals, educational or governmental institutions are rarely used. Less than half the SPRs from pre-university 12 th grade students actually demonstrate what could be labelled as both correct and adequate referencing.

Secondly, almost all SPRs contain at least one image or figure. However, students frequently insert images or figures of insufficient quality: the images are blurred, the text in the figure is not readable or the figure contains a watermark. Furthermore, when inserting a figure, graph or table, students often do not employ automatic numbering and captions. We see that students fail to apply the formula editor correctly when inserting a calculation in more than 85% of the cases. Both quantities and units are automatically displayed in italics. Students applying DRS correctly would put units upright. Other struggles we have encountered are the insertion of symbols, which is also often avoided or written in full text.

Empirical data collection is not present in all SPRs in the sample. Furthermore, less than a third of SPRs demonstrate the use of automated measurement via computer. Additionally, digital measurements are not always necessary in an SPR. In 40% of the SPRs, students gather data manually, e.g., a titration, analogue voltmeter. We see that students lack skills in using spreadsheets, e.g., calculation, processing and displaying data. This problem has been noted earlier by Bobkowski and Etheridge (2023), McHugh (2021) and Calvani (2012). This lack among secondary science students can lead to confusion and misinterpretation of research findings, which is similar to the findings of Guillén-Gámez (2020) among higher education teachers. Concretely, this means graphs lacking complete axis titles, secondary grid lines, a title above or showing a line from measurement point to measurement point. Furthermore, error bars have been used very infrequently in reports. We found that students do not regularly apply a slope of a curve correctly even when using a spreadsheet to construct diagrams digitally, they still use two points instead of points of the best-fit line. This is in line with Forster’s (2004) findings.

All this is especially noteworthy since all science examination programs in the Netherlands explicitly demand that students master skills to gather, analyse and visualise data digitally.

Finally, the skills to ‘Determine the accuracy and validity of sources/methods’ are only partly mastered. We see that pre-university students are able to use citing and paraphrasing. However, the correct notation of sources in a reference list is often found wanting. Again: the correct use of a list of references is a research skill, not a digital research skill, but automated notation can be readily distinguished from manual editing by scrutinizing the actual notation. The automated notation is considered to be a digital research skill.

### Limitations

The main limitation of this study is that the sample of SPRs was taken exclusively from Dutch students. The sample contained SPRs from schools throughout the country. The general needs cited in the introduction showed that the issues addressed are encountered in other countries as well. The collection of the SPRs was on a voluntary basis. Teachers were asked to submit all SPRs of their students made in the last year but may have chosen to submit only high-quality reports or students with a low-quality report might have refused to give their consent. We realise that this may be a source of bias towards selecting more high-quality reports. If so, this would mean the deficiencies in DRS would be even more problematic. In addition, not all DRS categories were present in the reports. For example, in a small number of SPRs, the obtained data were not processed into a graph. Therefore, we do not know whether these students are able to process the data into proper graphs. In this study, we can only code what is visible in an SPR. The process toward the SPR is not visible and no distinction can be made on whether a student cannot apply the skill or whether they are just careless.

### Implications

Our results show that some DRS are well-mastered and applied in pre-university science education. However, and this is consistent with research on the development of research skills and digital literacy skills in secondary education, the level at which DRS are applied in secondary education is often found wanting (Abdullah et al., 2011; Blankendaal-Tran et al., 2023; Erstad et al., 2021; Polizzi, 2020; Williams & Beam, 2019). For example, students in our study demonstrate the substandard use of essential functions in a word processor or a spreadsheet.

In line with the implications by McHugh et al. (2021), a possible way to improve science performance might be to make DRS an integral part of science education from early grades onwards. Whenever students engage in science projects and write reports about these, however small, they can be supported in and assessed on their level of DRS.

In a natural way, secondary school science students could then become familiar with: (1) the use and correct notation of (scientific) sources, (2) inserting images, graphs or tables using automatic numbering and adding a caption (3) the use of software with calculation function (4) the use of a formula editor, quantities and units, (5) the application of formatting options such as styles and headings.

To achieve this, teachers could be supported by specific tools, aligning with Guillén-Gámez’s (2020) emphasis on developing procedural and cognitive skills in teaching staff. These tools could include assessment instruments, with formative assessments providing ongoing feedback for real-time instructional adjustments, with regular summative assessments evaluating students’ overall progress. In addition, practical strategies such as using rubrics to set clear expectations and incorporating peer reviews to promote collaborative learning can be helpful. Teaching guidelines and examples of good practices provide ongoing feedback that helps teachers identify students’ strengths and weaknesses in DRS throughout the learning process. As a result, the level of knowing and applying DRS may rise and become suitable for a smooth entry into further (academic) education.

### Future Research

By using the DRS rubric as assessment tool, we have taken a concrete step forward bridging the gap in the transition to an academic science study. The rubric can be used as basis for a DRS tool for teachers and students, from undergraduates to the highest level in secondary education.

A follow-up study could focus on the development and evaluation of a digital guidance tool that helps teachers implement targeted interventions and improve their students’ DRS. This requires an inventory of teachers’ needs, a handbook and evaluation of that handbook. The tool should be concrete, easy to understand and accessible to both students and teachers. The DRS tool may have cross-curricular usefulness in secondary education, for instance in subjects such as mathematics and geography, where data from surveys may be analysed and visualised. Finally, this DRS tool would contribute to an overview with concrete demands and desired levels around SPRs, at least in the Netherlands.

Future studies could also focus on teachers’ needs to guide students in the DRS development, which is in line with the recommendations of Williams and Beam (2019), who advocated to identify how teachers can use digital tools to improve writing instruction, to meet the objectives of the writing curriculum, and engage all students in the composing process. Additionally, in this study we focused on the more technical competencies of DRS. However, the ethics of using digital technology, such as data management, should also be taken into consideration. Moreover, when the students’ reports were collected, their research had already been conducted. Analysing SPRs may not be the most suitable tool for addressing this aspect.

## Conclusion

This study shows that all graduating pre-university science students demonstrate DRS in their SPR, but to a limited extent. The students demonstrate skills in searching for a sufficient number of sources, referring to sources, displaying tables, selecting a diagram, adding page numbering and inserting a table of contents. However, the level of DRS as evidenced in their SPRs is wanting. Observed deficiencies of DRS in SPRs can lead to serious confusion: Because DRS are missing during the use of a spreadsheet, the obtained data are not presented properly; due to the lack of DRS when using a word processor, the structure of the text in the research paper can be confusing and if formatting functions are not well mastered, results may be misinterpreted. The results warrant further studies on the specific needs of both secondary school science teachers and students in supporting the development of digital research skills.

## Data Availability

The datasets used and/or analysed during the current study are available from the corresponding author on reasonable request.

## Appendix

Table 2

**Table 2:** Rubric for DRS, divided in 5 categories and 37 subcategories with three mastery levels and associated criteria according to ascending level. *Mandatory items during assessing

	DRS category	DRS subcategory skill	score 2	score 1	score 0
1	Browse, search and filter information	Number of digital resources *	sufficient to support the line of reasoning, e.g. > 5	almost sufficient to support line of reasoning, e.g. < 5	insufficient to support the line of reasoning, e.g. < 3
		Use of scientific sources *	majority from peer reviewed international journals and possibly educational/governmental institutions	at least 1 from peer-reviewed international journals and some scholarly pages/Wikipedia/etc	no peer-reviewed scientific articles, mainly blogs/scholarly pages/Wikipedia/interest groups
2	Gather, measure and collect digital content/data	Digital measuring tools	automated measurement via computer, e.g. online survey/simulation/sensors/applet/Arduino	computer is used, but measurement is not automated, e.g. online video interview via computer	computer is used during measuring, however incorrect.
3	Determine the accuracy and validity of sources	Referring, quoting and paraphrasing*	correctly applied, e.g. plagiarism < 10%	almost always applied, sometimes referred to sources in text	incorrectly applied, e.g. no reference to sources in text
5	Analyse, transform and visualise content/data	Digitally processing large amounts of data	> 50 measuring points obtained digitally	between 10 and 50 measurement points obtained digitally	fewer than 10 measuring points digitally acquired
		Selection diagram	the type of diagram fits the data	almost right, e.g. the graphs could be combined into one graph	type does not fit the data, e.g. would have chosen a pie chart instead of a scatter diagram
		Display data from different types of measurements	uses multiple types of diagrams (pie chart, bar chart)	one type of diagram used	data not processed into a diagram, while needed.
		Using software with calculation function	uses advanced software, e.g. Excel, Python	software used, e.g. COACH, Tinkercad	no software used, e.g. manual/Google Forms
		Multiple data sets displayed in one diagram	correct, e.g. clear difference between different series	almost correct, e.g. difference between different ranges not clear when printing in black and white	incorrect, e.g. unclear difference between different series
		Display graph	correct, e.g. with grid lines and measuring points	almost correct, e.g. does not contain grid lines, inconsistent with significance axis values	incorrect, e.g. contains only a line, no measurement points
		Axis titles	correct, e.g. with correct quantities and units	almost correct, e.g. correct quantities but no/incorrect units, weight(grams)	incorrect or missing, e.g. both quantity and unit missing
		Trend line	right, e.g. right type of trend line through the measurement points	almost correct, e.g. the trend line should have passed through the origin, formula trend line without correct quantities	incorrect, e.g. line drawn from measurement point to measurement point
		Legend	correct, e.g. categories/series clearly and completely explained	almost correct, e.g. explanation legend incomplete, e.g. about the ranges/categories/+, ++-, +-, --+, -	no legend while needed or not needed and present, explanation missing
		Error bars	correct	almost correct	incorrect
		Formatting graph	correct	almost correct, e.g. contains an outline	incorrect, e.g. contains a title above the graph, menu bar COACH visible
		Display table	correct, e.g. column headings correct and dates clear	almost correct, e.g. data missing, significance not set or contains magnitudes but no units, 0.00000632 instead of scientific notation	incorrect, e.g. unclearly displayed making it non-functional.
		Formatting table	correct, e.g. columns/rows neatly spaced and text aligned	almost correct, e.g. irregularities in design, e.g. row contains an extra white line, column heading not distinguished	incorrect, e.g. table not formatted, columns/rows not in proportion, text not aligned
		Display pictures/images	correct, e.g. sharp and clearly visible	almost correct, e.g. too small/too dark	incorrect, e.g. vague, contains a watermark, English terms
		Reaction equations	correct, e.g. using formula editor	almost correct, e.g. indexes and coefficients also italicised, --> instead of	incorrect, e.g. everything in italics or handwritten and scanned in
		Drawing structural formulas	correct	almost correct, e.g. binding lines not aligned	incorrect, e.g. hand-drawn and scanned or missing while needed
		Programming/Technical drawing	code largely self-written/drawn	code largely not self-written/drawn	code not written/signed or creation of code not indicated
6	Write a research paper using digital tools	Page numbering *	correct, e.g. automatic	almost correct, e.g. not updated	incorrect, e.g. manual
		Images/figures/tables numbering	correct, e.g. automatic	almost correct, e.g. manually, at figure above, at table below	incorrect, e.g. not present
		Table of contents *	correct, e.g. automatic	almost correct, e.g. not updated, >4 levels of subheads, empty lines with page no.	incorrect, e.g. manual, not present
		Insert captions *	correct, e.g. automatic insertion function used everywhere	almost correct, e.g. not automatic insertion function universally	not present or incorrect, e.g. automatic insertion function not used
		Reference list *	correct, e.g. according to APA/IEEE	almost correct, e.g. URL database instead of URL journal	incorrect, e.g. no logical sequence, information missing, as endnotes/single URL’s in a row
		Styles and headings *	correct, e.g. consistently and automatically used everywhere	almost correct, e.g. not consistent, subheads not relevant	incorrectly or manually applied
		(Non) Italics	correctly applied	almost correct/constantly applied	incorrect, e.g. quantities not in italics
		Sub-/Superscript	correctly applied	applied almost correctly/consequently, e.g. x^3	incorrect or not applied to indexes/coefficients/loading
		Symbols	correctly applied	almost correctly/consequently applied, e.g. * instead of ∙	incorrect, e.g. labda/L instead of λ or C instead of ⁰C
		Listings	correctly applied	applied almost correctly/constantly, e.g. different symbols/insertions all the time	incorrect, e.g. listing 1 line, not applied while needed
		Line spacing *	correctly applied	applied almost correctly/constantly, e.g. different distances between 2 paragraphs/objects	incorrect, e.g. different distances within the same paragraph
		Text wrapping *	correct, e.g. objects in text line or clearly in a column next to the text	almost correct, e.g. objects not always in text line, but to left or right of text	incorrect, e.g. arrangement images irregular/on top of each other
		Layout, alignment and margins *	correct, e.g. consistent use of 2 columns, margins/indentation consistent	almost correct, e.g. margins/indentation not consistent, alternating 1 and 2 columns in the same paragraph	incorrect, e.g. table ends over the edge of the page
		Header and footer	correct, e.g. recurring title/image/colour in header	almost correct, e.g. not consistently applied	incorrect
		Representation research design	correct, e.g. arrangement displayed digitally in a drawing or diagram	almost correct, e.g. only represented by a photo	incorrect, not reproduced or hand-drawn
		Format formula/calculation	correct, e.g. use of formula editor	almost correct, e.g. magnitudes not italicised and/or text/numbers not used upright/* instead of ∙	incorrect or manual, e.g. fractions or powers not applied with correct function, *v* = Δ*x*/Δ*t*

Table 3

**Table 3:** Results per category of the DRS framework. *Mandatory items during assessing

	DRS category	DRS subcategory skill	
1	Browse, search and filter information	Number of digital resources *	79 out of 88 SPRs showed the use of an adequate amount of digital resources to support the line of reasoning.
		Use of scientific sources *	4 out of 88 SPRs contain scientific peer-reviewed sources in more than half of the references, others mainly use internet sources, sometimes of questionable quality.
2	Gather, measure and collect digital content/data	Digital measuring tools	25 out of 47 SPRs used automated digital measurements, others were manually obtained (even though they may have used a computer, e.g., mass, current, force).
3	Determine the accuracy and validity of sources	Referring, quoting and paraphrasing *	65 out of 88 SPRs refer to sources in the main text, a small number did not refer to sources in the text, use quotation or paraphrasing.
5	Analyse, transform and visualise content/data	Digitally processing large amounts of data	21 out of 60 SPRs process more than 50 digitally obtained measuring points, others contain fewer than 10 digitally acquired measuring points
		Selection diagram	36 out of 44 SPRs contain a type of diagram that fits the data, others chose a pie or bar chart instead of a scatter diagram.
		Display data from different types of measurements	12 out of 50 SPRs use more than one type of diagram, others mainly use the same type of diagram or many tables to display data of different types of measurements.
		Using software with calculation function	28 out of 60 SPRs used Microsoft Excel to process data, in 1 SPR Python is used for calculation functions.
		Multiple data sets displayed in one diagram	14 out of 19 SPRs displayed multiple data sets correctly in one diagram, others show each measurement in its own diagram.
		Display graph	In 19 out of 45 SPRs the graphs were correctly displayed, including measuring points and grid lines, others contain a line without measurement points.
		Axis titles	In 17 out of 42 SPRs graph axis titles were correctly displayed, others had a missing quantity and/or unit.
		Trend line	In 4 out of 13 SPRs the graphs show the correct type of trend line through the measuring points, others showed that the trend line did not cross the origin when it was expected to do so.
		Legend	19 out of 23 SPRs show a clear and completely explained legend.
		Error bars	2 out of 2 SPRs show correctly applied error bars in their graph.
		Formatting graph	7 out of 45 SPRs show correctly formatted graphs, others did not remove default elements added by Excel.
		Display table	42 out of 58 SPRs contain correctly displayed tables, others did not have clear and correct headings.
		Formatting table	21 out of 58 SPRs contain correctly formatted tables, others did not have neatly spaced columns/rows and aligned text.
		Display pictures/images	25 out of 83 SPRs contain sharp and clearly visible images, some others contain watermarked images.
		Reaction equations	3 out of 19 SPRs show digitally correct reaction equations, some others had taken a picture of a handwritten reaction equation.
		Drawing structural formulas	0 out of 1 SPRs show a self-drawn digital structural formula
		Programming/Technical drawing	5 out of 8 SPRs show self-programmed code or self-drawn digital design, others had partially programmed/drawn this themselves.
6	Write a research paper using digital tools	Page numbering *	83 out of 88 SPRs include the use of automatic page numbering
		Images/figures/tables numbering	29 out of 86 SPRs include the use of automatic figure/table/image numbering
		Table of contents *	58 out of 88 SPRs include the use of an automatic table of contents
		Insert captions *	39 out of 88 SPRs captions are consistently inserted correctly
		Reference list *	36 out of 88 SPRs showed correctly applied notation (APA/IEEE) styles, others mainly use a list of URLs.
		Styles and headings *	26 out of 88 SPRs show the use of automatic styles and headings.
		(Non) Italics	8 out of 38 SPRs contain correct use of italics, e.g. quantities.
		Sub-/Superscript	30 out of 60 SPRs contain correct use of sub- and superscript, e.g. formulas.
		Symbols	23 out of 43 SPRs contain correct use of symbols, e.g. quantities and units.
		Listings	40 out of 81 SPRs contain correct use of listings.
		Line spacing *	34 out of 88 SPRs contain correct use of line spacing, others are mainly inconsistent.
		Text wrapping *	22 out of 88 SPRs show the correct use of text wrapping, some others arrangement images irregular across the page.
		Layout, alignment and margins *	42 out of 88 SPRs show the correct use of alignment and margins, others are mainly inconsistent.
		Header and footer	8 out of 10 SPRs show the correct use of headers and footers.
		Representation research design	11 out of 47 SPRs contain a digital representation of the research design, others contained no representation or were hand-drawn.
		Format formula/calculation	5 out of 40 SPRs contain the correct representation of formulas, e.g. use of a formula editor.
